# Exploration of the Crucial Genes and Molecular Mechanisms Mediating Atherosclerosis and Abnormal Endothelial Shear Stress

**DOI:** 10.1155/2022/6306845

**Published:** 2022-08-12

**Authors:** Guoqi Zhu, Yan Lai, Fei Chen, Jun Qian, Hao Lin, Deqiang Yuan, Tongqing Yao, XueBo Liu

**Affiliations:** Department of Cardiology, Tongji Hospital, School of Medicine, Tongji University, Shanghai, China

## Abstract

**Background:**

Abnormal endothelial shear stress (ESS) is a significant risk factor for atherosclerosis (AS); however, the genes and pathways between ESS and AS are poorly understood. Here, we screened hub genes and potential regulatory targets linked to the progression of AS induced by abnormal ESS.

**Methods:**

The microarray data of ESS and AS were downloaded from the Gene Expression Omnibus (GEO) database. The coexpression modules related to shear stress and AS were identified with weighted gene coexpression network analysis (WGCNA). Coexpression genes in modules obtained from GSE28829 and GSE160611 were considered as SET1. The results were validated in validation set by differential gene analysis. The limma package in R was used to identify differentially expressed genes (DEGs). The common DEGs of GSE100927 and GSE103672 were regarded as SET2. Next, Gene Ontology (GO) annotation and Kyoto Encyclopedia of Genes and Genomes (KEGG) pathway enrichment analysis was conducted. Protein-protein interaction (PPI) enrichment analysis was assembled, and hub genes were identified using MCODE and ClueGO in Cytoscape. ROC curve analyses were conducted to assess the ability of common hub genes to distinguish samples of atherosclerotic plaque from normal arterial. The expression of common hub gene was verified in ox-LDL-induced foam cells and GSE41571.

**Results:**

We identified three gene modules (the blue, tan, and cyan modules) related to AS and three shear stress-related modules (the brown, red, and pink modules). A total of 129 genes in SET1 and 476 genes in SET2 were identified. *CCRL2*, *LGALS9*, and *PLCB2* were identified as common hub genes and validated in the GSE100927, GSE28829, and GSE41571. ROC analysis indicates the expression of *CCRL2*, *LGALS9*, and *PLCB2* could effectively distinguish the atherosclerotic plaque and normal arterial. The expression level of *CCRL2*, *LGALS9*, and *PLCB2* increases with the accumulation of lipid increased.

**Conclusion:**

We identified *CCRL2*, *LGALS9*, and *PLCB2* as key genes associated with abnormal ESS and AS and may provide potential prevention and treatment target of AS induced by abnormal ESS.

## 1. Introduction

Atherosclerosis (AS) and its complications are the leading cause of death and disability in the world and China [1, 2]. AS is a long-term chronic inflammatory disease, characterized by subintimal lipid deposition, endothelial injury, inflammatory cell infiltration, and atherosclerotic plaque formation [3]. However, the current study cannot fully explain the pathogenesis of AS, which needs further exploration and research. The endothelial cells (ECs), a primary layer of protection for vascular, is constantly exposed to a variety of stimuli and insults from circulation [4]. The entire vasculature is exposed to atherosclerotic risk factors, such as hyperglycemia, inflammatory cell infiltration, and abnormal blood flow shear stress, which promote the progress of AS by inducing endothelial dysfunction, but atherosclerotic plaques tend to form and progress in specific areas of arteries where disordered flow leads to abnormal endothelial shear stress (ESS) [5–7].

ESS is a kind of tangential stress generated by the friction of flowing blood upon the endothelial surface of blood vessels, which depends on blood viscosity and velocity gradient at the wall and regulates many functions of endothelium [8]. There are two main blood flow patterns, oscillatory shear (OS) observed at branch points and pulsatile shear (PS) prevalent in straight segments of arteries. OS promotes the endothelial atherogenic phenotype, while PS is associated with an atheroprotective endothelial phenotype [9]. It has long been appreciated that OS induced by blood flow is known as the risk factor with great contribution to the development of AS [10]. By acting on ECs, abnormal blood flow causes OS to activate ECs, resulting in the release of inflammatory factors, and then endothelial dysfunction, which is a significant contribution in the subclinical stages of AS [11, 12].

Several studies have shown that OS could induce widespread gene expression alterations in ECS that might be involved in the progression of AS [8, 13–15]. Studies have demonstrated that vulnerable atherosclerotic plaques preferentially develop in regions with OS, and the fibrous cap was thinner, and the prevalence rates of thin-cap fibroatheroma (TCFA) were higher in the vascular segments with persistently OS than in other segments [7, 16]. Although the close link between OS and AS plaque formation has been recognized, the intimate molecular mechanisms remain unclear.

Detection of gene expression by high-throughput sequencing technology is a very powerful tool to reveal the potential genes and biological mechanisms in the process of atherosclerotic plaque formation, which provides a new direction for the discovery of cardiovascular disease mechanism [17]. In this study, bioinformatic technology was used to analyze the relationship between pulsatile or oscillatory ESS and AS, in order to determine the common molecular mechanism of AS and ESS. This approach is particularly useful for revealing the master regulatory or hub genes identified in the differential coexpression network since the hub genes are expected to play a key role in regulating the expression of dozens of other genes in the network. That may provide potential targets for the prevention and treatment of AS.

## 2. Materials and Methods

### 2.1. Microarray Datasets

**Figure 1:**
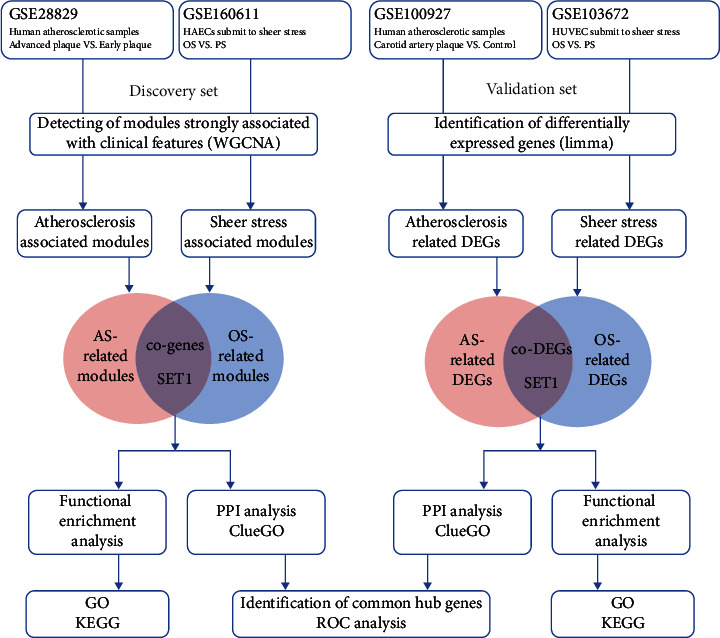
Workflow of bioinformatics analysis.

The GEO database (https://www.ncbi.nlm.nih.gov/geo), fully known as gene expression omnibus, incorporates high-throughput gene expression data proposed by global research institutions [18]. To explore the effects of abnormal blood flow shear forces on ECs, we screened in the GEO database according to the following conditions: first, the control group must contain a control group and experimental groups, and the experimental group must contain different blood flow shear forces. Second, cells used for used for experiments and sequencing should be ECs. To analyze gene expression changes in atherosclerotic plaques, the enrolled datasets had to contain atherosclerotic plaque tissues and corresponding control tissues; all samples must be derived from carotid artery tissues. In addition, these datasets must provide the original data for our reanalysis. In the present research, to investigate the relationship between pulsatile or oscillatory ESS and AS, we searched the GEO database and selected datasets GS160611, GSE103672, GSE28829, and GSE100927. GSE160611 contains the gene expression dataset of human aortic endothelial cells (HAECs) submitted to PS and OS flows. PS and OS flows were applied to ECs with shear stresses of 12 ± 4 dyn/cm^2^ and 0.5 ± 4 dyn/cm^2^, respectively. For static condition (ST), samples were collected at 0 hour under no flow [9]. Samples for RNA sequencing analysis were collected at 1, 4, and 24 hours after exposure to shear, with three biological replicates for each experimental condition. In GSE103672, PS (12 ± 5 dyn/cm^2^) or OS (0.5 ± 5 dyn/cm^2^) was applied to human umbilical vein endothelial cells (HUVECs), and samples were collected at 1, 2, 3, 4, 6, 9, 12, 16, 20, and 24 hours after exposure to shear, and two replicates were used for each condition/time point [19]. GSE100927 contains the gene expression dataset of 29 atherosclerotic carotid arteries and 12 control arteries [20]. The GSE28829 dataset consists of 13 early and 16 advanced human carotid atherosclerotic plaque samples [21]. In GSE41571, genome-wide gene expression profiling was performed on macrophage-rich regions of 6 stable and 5 ruptured human atheromatous plaques derived from carotid endarterectomy samples. GSE41571 was used to validate the differential expression of hub genes from ruptured and stable atherosclerotic human plaques. Based on the workflow, we analyzed the five datasets and screened and verified hub genes (Figure 1).

### 2.2. Coexpression Network Construction with WGCNA

Weighted gene coexpression network analysis (WGCNA), as an effective method for detection and exploration of deep relationships between genes and diseases, allows the identification of the gene coexpression modules and genes with high connectivity within the modules by using a hierarchical clustering approach [22]. Datasets were downloaded from the NCBI-GEO public database, and the “WGCNA” package in R studio software was employed to obtain modules associated with both advanced plaque samples and early plaque samples in GSE28829, and for GSE160611, WGCNA was carried out to find modules highly correlated with PS and OS. The first 10000 genes with large variation were involved in further analysis. The “Hclust” function in R studio software was used to perform clustering analysis for excluding the outlier samples, and the “pickSoftThreshold” function in the “WGCNA” package, according to the standard of scale-free network, was used to calculate appropriate soft power *β* for further matrix construction, using the soft power value and the formula *a*_*mn*_ = |*c*_*mn*_|^*β*^ (*a*_*mn*_ is the adjacency matrix between gene *m* and gene *n*, *c*_*mn*_ represents Pearson's correlation coefficient between gene *m* and gene *n*, and *β* is the soft power value) to create the weighted adjacency matrix. Based on an adjacency matrix, the topological overlap matrix and the corresponding dissimilarity were created for detecting gene module. The dendrogram was further divided into different gene expression modules, and the correlation between different gene expression modules was calculated. The genes in the modules closely related to clinical characteristics were selected for subsequent analysis. After selecting modules of interest, the plaque-associated module and the shear force-associated module were intersected to obtain the common genes.

### 2.3. Validation of Gene Expression through DEG Analysis

Differential gene expression was performed with limma package with |log FC| > 0.5 and adjusted *P* value < 0.05 [23]. From the GSE100927, we got the differentially expressed genes (DEGs) between carotid plaque and carotid control samples. For the GSE103672 dataset, the DEGs of OS (0.5 ± 5 dyn/cm^2^ 12, 16, 20, and 24 hours) compared with PS (12 ± 5 dyn/cm^2^ 12, 16, 20, and 24 hours) were identified. Both DEGs were introduced into the online tool (https://www.xiantao.love/products) to obtain common DEGs (co-DEGs) of GSE100927 and GSE103672.

### 2.4. Functional Enrichment Analysis

In order to reveal the underlying biological functions of genes related to shear stress and atherosclerotic plaque, common genes were imported into the DAVID database (https://david.ncifcrf.gov/) for Gene Ontology (GO) and Kyoto Encyclopedia of Genes and Genomes (KEGG) enrichment analyses [24]. GO is an international standardized gene functional classification system. GO terms were divided into three gene set libraries: biological process (BP), cellular component (CC), and molecular function (MF) [25, 26], and KEGG describes the pathways enriched in gene sets [27].

### 2.5. Identification of Hub Genes

Both common gene lists were loaded into the STRING database for protein-protein interaction (PPI) enrichment analysis [28]. According to the PPI network, the molecular complex detection (MCODE) algorithm in Cytoscape software has been applied to identify the densely connected subnetwork [29]. To explore potential roles of these genes in shear stress and AS, the ClueGO plug-in in the Cytoscape software was used for functional enrichment analysis and visualization of the cluster of interest. Clusters of two PPI network were intersected to get common hub genes.

### 2.6. Receiver Operating Characteristic (ROC) Curve for Diagnostic Effectiveness Evaluation of Common Hub Genes

The expression profile of common hub genes was acquired to visualize the expression profile of common hub genes in datasets GSE160611, GSE100927, GSE28829, and GSE41571. To evaluate the diagnostic effectiveness evaluation of common hub genes, the samples were divided into plaque and control groups. The pROC package and ggplot2 package were used to draw ROC curve and calculate the area under the curve (AUC) to evaluate the capability and sensitivity of common hub genes to distinguish atherosclerotic samples from control group [30, 31]. According to the previous studies, AUC = 0.5 indicates no evaluation efficacy, 0.7 ≤ AUC < 0.8 indicates acceptable evaluation efficacy, 0.8 ≤ AUC < 0.9 represents excellent evaluation efficacy, and AUC ≥ 0.9 means outstanding evaluation efficacy [32].

### 2.7. Validate the Expression of Common Hub Genes in Foam Cells

The human monocytic cell line (THP-1), purchased from American Type Culture Collection (ATCC), was grown in complete RPMI-1640 (supplemented with 10% fetal bovine serum, 1%penicillin/streptomycin, and 2 mM GlutaMAX). The cells were cultured at 37°C in 5% CO_2_ and subcultured at 80–90% confluence. THP-1 cells were differentiated into macrophages with a dose of PMA (Merck) at 100 ng/ml for 72 hours. To induce foam cell formation, the macrophages were incubated with 25 or 50 *μ*g/ml ox-LDL for 24 hours. Foam cells were assessed by Oil Red O Staining kit (Beyotime, C0158S, China).

### 2.8. Real-Time Quantitative PCR Assay

RNA was extracted from foam cells using the RNeasy kit (Qiagen, 74104, Germany) according to manufacturer's instructions. Total RNA (500 ng) from foam cells was reverse-transcribed into cDNA using PrimeScript RT Master Mix (TaKaRa, RR036A, China). The real-time quantitative PCR reaction was performed on the QuantStudio™ 5 system (Thermo Fisher Scientific, USA) using qPCR SYBR Green Master Mix (Vazyme, Q141-02, China). PCR was performed by the following conditions: hold stage: 95°C for 30 s, PCR stage: 40 cycles at 95°C for 5 s and 60°C for 30 s, and melt curve stage: 95°C for 15 s and 60°C for 60 s. The Ct values were normalized using the housekeeping gene GAPDH, and the mRNA expression levels were calculated as the 2^-*ΔΔ*Ct^ value. Primers used for real-time quantitative PCR were included in Supplementary Table 5.

## 3. Results

### 3.1. GEO Dataset Information

We selected four GEO datasets for analysis; datasets GSE100927 and GSE28829 contain the sequencing results of atherosclerotic plaque samples; GSE160611 and GSE103672 contain flow shear force-induced sequencing data for endothelial cell samples. For WGCNA analysis, we selected GSE28829 and GSE160611 as discovery set. GSE100927 and GSE103672 were paired as validation sets for DEG analysis (Table 1). A schematic diagram of our workflow is shown in Figure 1.

### 3.2. The Coexpression Modules Related to Atherosclerotic Plaque and Shear Stress in Discovery Set

**Figure 2:**
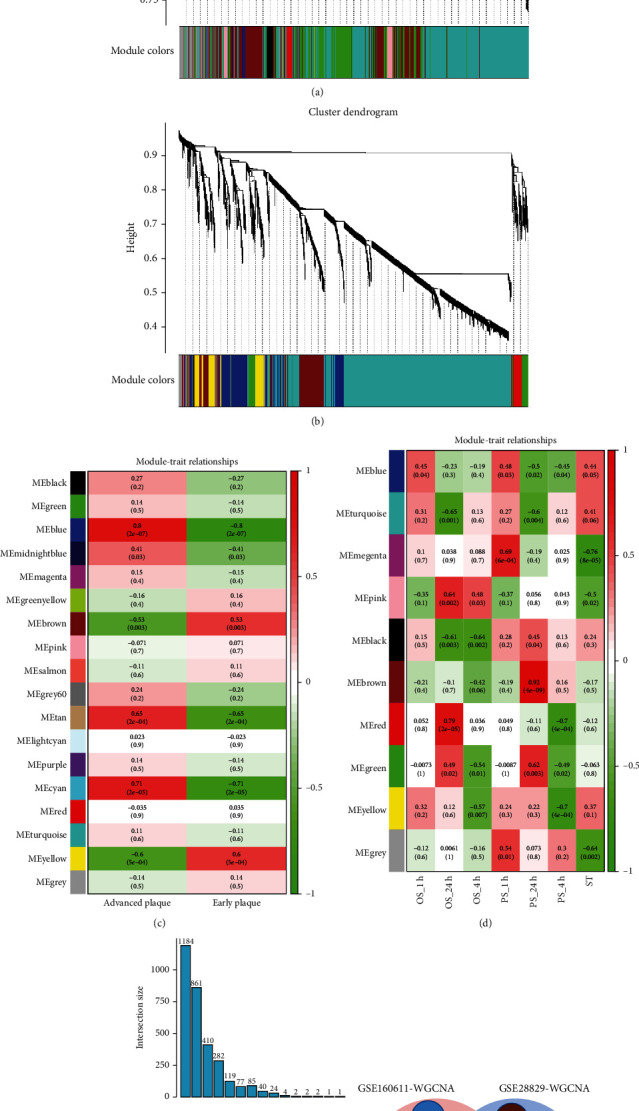
Weighted gene coexpression network analysis (WGCNA). (a) The cluster dendrogram of coexpression genes in GSE28829. (b) The cluster dendrogram of coexpression genes in GSE160611. (c) Module-trait relationships in atherosclerosis. Each cell contains the corresponding correlation and *p* value. (d) Module-trait relationships in shear stress. Each cell contains the corresponding correlation and *p* value. (e) The left bar graph demonstrates the total amount of genes contained in each module. The points on the bottom right refer to the corresponding dataset names on the left side by their correspondences in the horizontal direction. The bar chart at the top right indicates the number of intersection genes. (f) The shared genes (SET1) between the modules of GSE28829 and GSE160611 by overlapping them.

We performed a WGCNA of discovery set and identified a series of gene modules associated with atherosclerotic plaque and shear stress. For GSE28829, WGCNA identified modules that were highly correlated with atherosclerotic plaque by plotting a heatmap of module-trait relationships, with each color representing a specific module (Figures 2(a) and 2(c)). Among the 18 modules identified, three modules “blue,” “cyan,” and “tan” exhibited significant positive correlations with atherosclerotic plaques (blue module: *r* = 0.80, *p* = 2e − 07; cyan module: *r* = 0.71, *p* = 2e − 05; and tan module: *r* = 0.65, *p* = 2e − 04) and were selected for further analysis (Figures 2(a) and 2(c)).

There were 1184, 77, 119 genes in the blue, cyan, and tan modules, respectively (Figure 2(e)). Similarly, WGCNA identified 10 modules of coexpressed genes in GSE160611 (Figures 2(b) and 2(d)). Of the 10 modules of highly correlated genes identified in this analysis, red modules (*r* = 0.65, *p* = 2e − 04) were positively correlated with OS (exposure to OS shear for 24 h). With the increase of stimulation time, the positive correlation between pink module (exposure to OS shear for 24 h: *r* = 0.64, *p* = 0.002) and OS stimulation gradually increased. The brown module (*r* = 0.92, *p* = 4e − 09) was positive correlated with PS (exposure to PS shear for 24 h). There were 410, 282, 861 genes in the red, pink, and brown modules, respectively (Figure 2(e)). Figure 2(e) shows the number of genes in the individual modules and their intersection by pairwise. The 129 overlapped genes of modules obtained from GSE28829 and GSE160611 were identified as SET1 (Figures 2(e) and 2(f) and Supplementary Table 1).

### 3.3. Differentially Expressed Genes Associated with Atherosclerotic Plaque and OS in Validation Set

**Figure 3:**
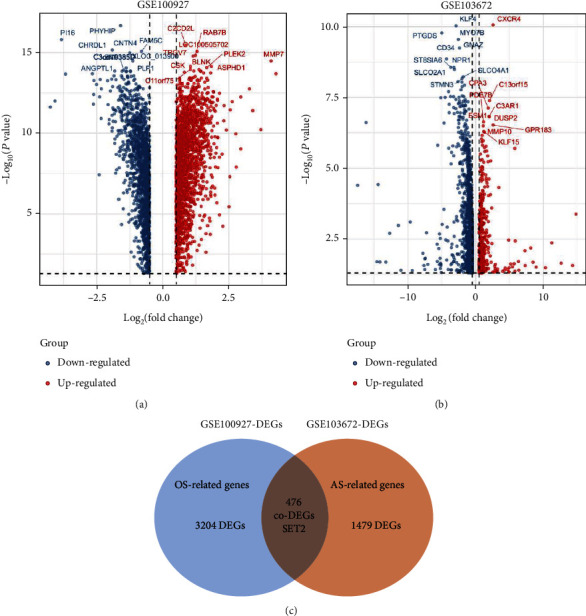
Identification of differential expressed genes (DEGs). (a) Volcanic plot of 3680 DEGs from GSE100927. We got the DEGs between plaque and control samples The *x*-axis represents the log2 (fold change) and the *y*-axis represents the *p* value. Blue to red color dots represents low to high expression level. (b) Volcanic map of 1955 DEGs of HUVECs under oscillatory shear stress compared to those in physiological shear stress samples in GSE103672 dataset. (c) Venn diagrams showing the 476 overlapped genes in the GSE100927 and GSE103672 datasets, which were defined as SET2.

In the present research, we searched the GEO database and selected datasets GSE100927 and GSE103672 to identify DEGs. GSE100927 contained samples of atherosclerotic lesions and control arteries without atherosclerotic lesions. 1955 DEGs were identified from carotid atherosclerotic plaque samples based on the gene expression of control group, which were considered as AS-related genes (Figures 3(a) and 3(c)). We identified 3680 DEGs in OS (0.5 ± 5 dyn/cm^2^) compared with physiological ESS (12 ± 5 dyn/cm^2^) from GSE103672, and these DEGs were regarded as OS-related genes (Figures 3(b) and 3(c)). A total 476 intersection genes were selected as SET2 from both AS-related genes and OS-related genes (Figure 3(c) and Supplementary Table 1).

### 3.4. Functional Enrichment and Pathway Analysis

**Figure 4:**
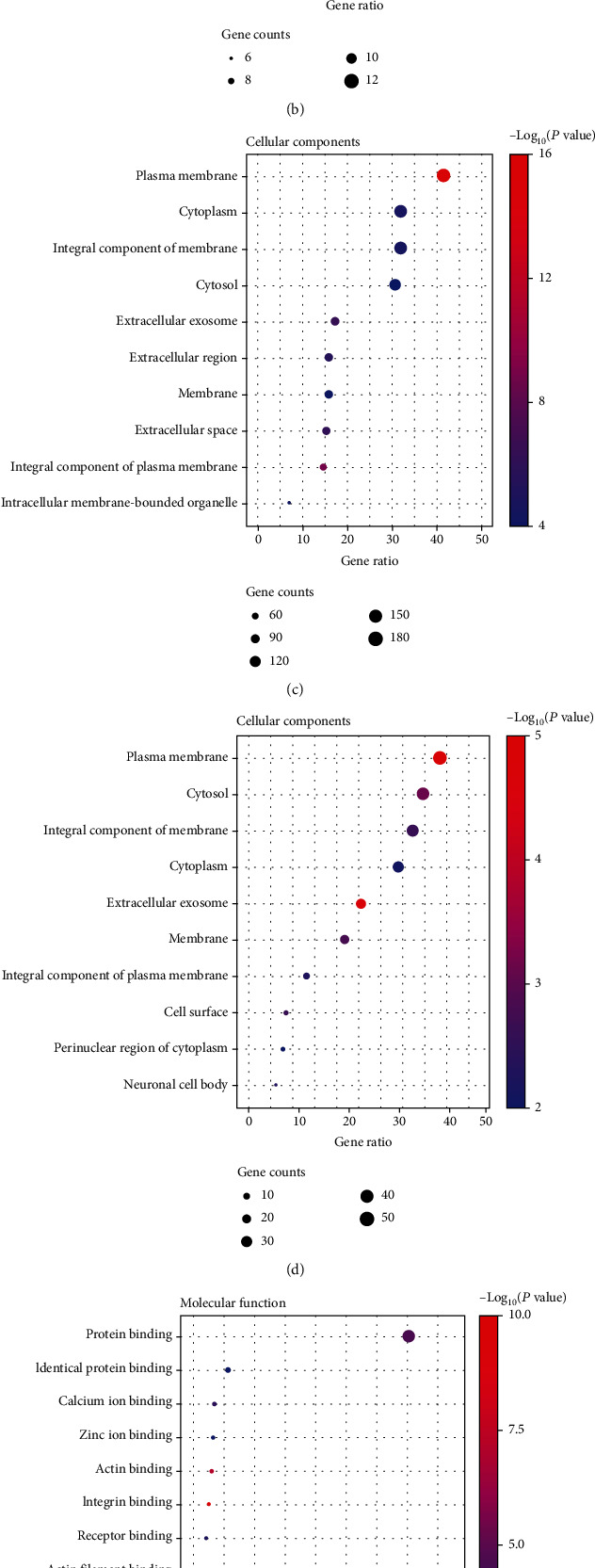
Gene Ontology (GO) analyses of SET1 and SET2 in the biological processes (BPs), cellular components (CCs), and molecular functions (MFs). (a) BP analyses of SET1. Bubble plot of GO: the *x*-axis represents the ratio of term genes to the total genes, and *y*-axis represents the pathway term name. The size of dots represents the number of genes associated with the GO term, and the color corresponds to the *p* value. (b) BP analyses of SET2. (c) CC analyses of SET1. (d) CC analyses of SET2. (e) MF analyses of SET1. (f) MF analyses of SET2.

**Figure 5:**
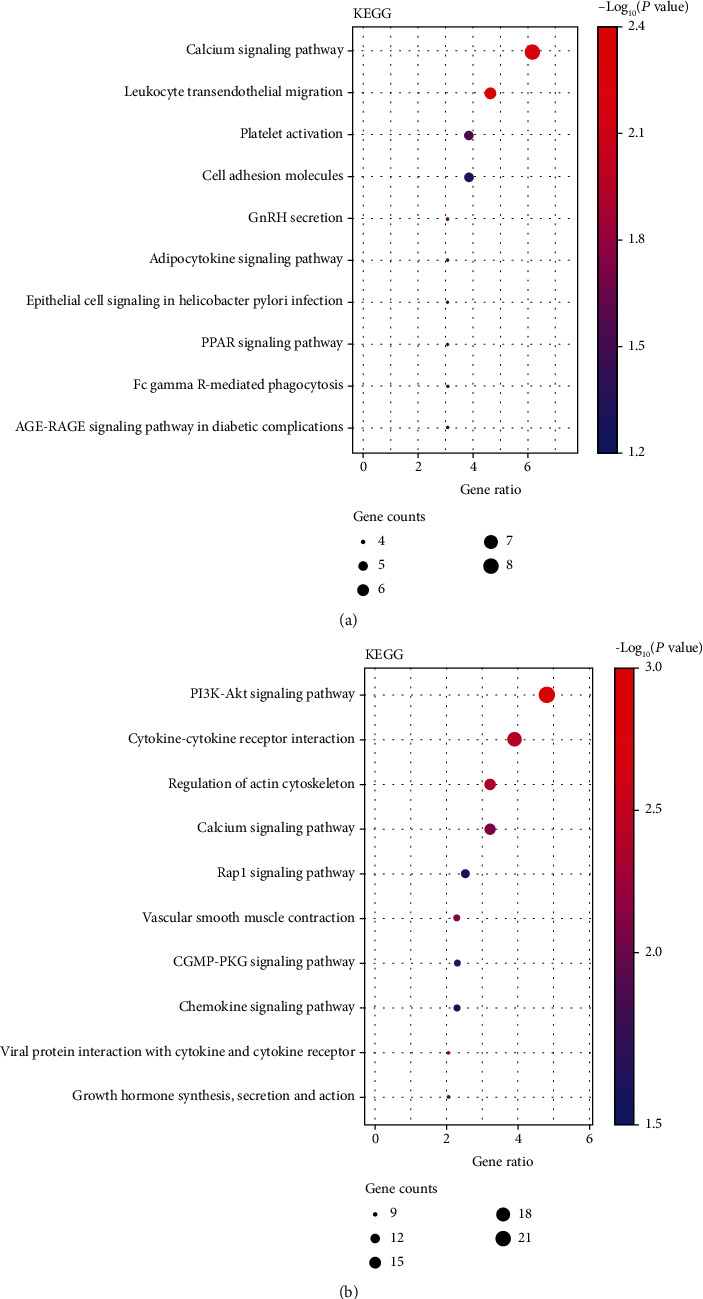
Kyoto Encyclopedia of Genes and Genomes (KEGG) analyses of SET1 and SET2. (a) Bubble plot of KEGG analyses of SET1. (b) Bubble plot of KEGG analyses of SET2.

GO and KEGG pathway analyses were performed by importing SET1 and SET2 obtained from discovery set and validation set to the DAVID online database. GO-BP, GO-CC, and GO-MF pathways with top 10 and top 20 gene counts of this module were selected to be shown in Figure 4 and Supplementary Table 2-3, respectively. Notably, functional enrichment analysis showed a highly significant similarity between the pathways enriched in the two gene sets (SET1 and SET2). For GO-BP, five items of “cell adhesion,” “intracellular signal transduction,” “inflammatory response,” “angiogenesis,” and “cytokine-mediated signaling pathway” are significantly enriched in both SET1 and SET2 (Figures 4(a) and 4(b)). Similarly, GO-CC analysis (Figures 4(c) and 4(d)) shows that a variety of terms are enriched exclusively in both SET1 and SET2 (“plasma membrane,” “cytosol,” “integral component of membrane,” “cytoplasm extracellular exosome,” “membrane,” and “integral component of plasma membrane”). SET1 and SET2 share “Protein binding” pathways in GO-CC module (Figures 4(e) and 4(f)). KEGG enrichment analysis also revealed genotype-specific enriched KEGG terms (Figures 5(a) and 5(b)). While the SET1 and SET2 were both enriched in “Calcium signaling pathway” terms, each gene set contained also a variety of specifically enriched KEGG terms.

### 3.5. Construction of Protein-Protein Interaction (PPI) Network and Identification of Hub Genes

After pathway enrichment analysis, PPI network was constructed, and hub genes were screened using Cytoscape software. In order to constructed PPI network, we introduced SET1 and SET2 into STRING databases, respectively, for PPI analysis. Both string interaction networks were imported to Cytoscape software, and the MCODE algorithm was applied to identify densely connected network components.

**Figure 6:**
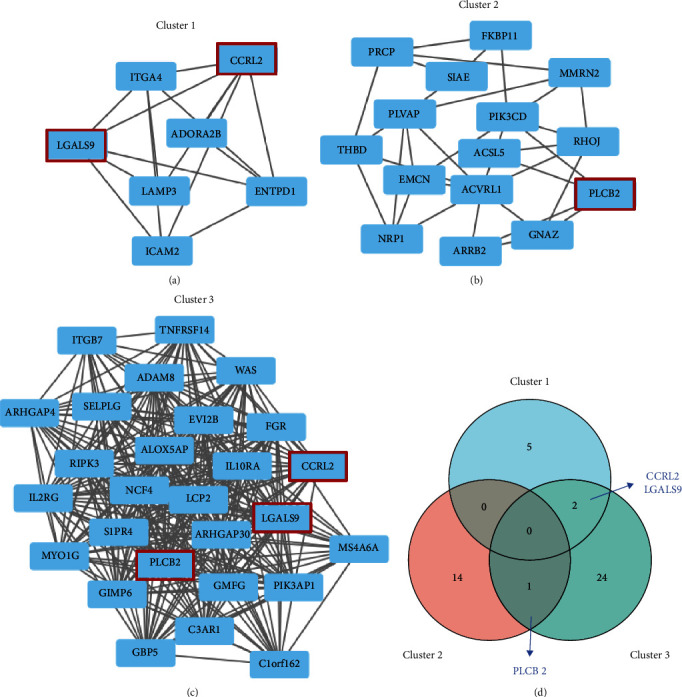
The PPI network and clusters analysis of SET1 and SET2. (a) The cluster was identified by Cytoscape MCODE algorithm from STT1. The dots represent the hub genes identified by the MCODE algorithm. (b) Another cluster identified from STT1. (c) The cluster identified from STT2. (d) The shared genes between the clusters.

For SET1, we got 22 hub genes in two densely connected gene cluster 1 and cluster 2 (Figures 6(a) and 6(b) and Supplementary Table 4), and 27 hub genes in cluster 3 were obtained from SET2 (Figure 6(c) and Supplementary Table 4). Significantly, *CCRL2* (C-C motif chemokine receptor-like 2), *LGALS9* (also known as galectin-9), and *PLCB2* (phospholipase C beta 2) were identified as common hub genes, indicating their key roles in shear stress and atherosclerotic plaque (Figure 6(d)).

**Figure 7:**
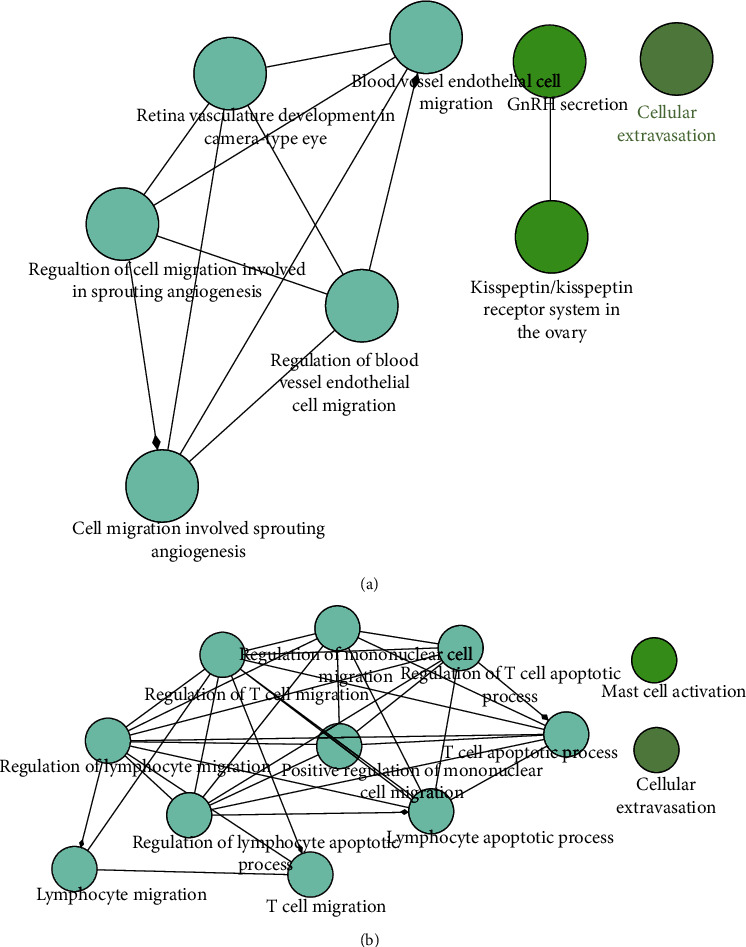
ClueGO enrichment analysis of three clusters. (a) The interaction network of GO terms of cluster 1 and cluster 2 generated by the Cytoscape plug-in ClueGO. The significant term of each group is highlighted. (b) The interaction network of GO terms of cluster 3.

We then explored the potential role of these genes in three clusters. ClueGO, a plug-in of Cytoscape that can be used to classify and visualize nonredundant GO terms as networks with functional groupings, was used here for biological analysis of these genes. Genes in cluster 1 and cluster 2 were combined for analysis, and they mainly focused on pathways related to “regulation of blood vessel endothelial cell migration” and “cell migration involved in sprouting angiogenesis” (Figure 7(a)). Cluster 3 was mainly related to “positive regulation of mononuclear cell migration” and “T cell migration” (Figure 7(a)). As a whole, these genes in three clusters are predominately involved in cell migration.

### 3.6. Validation of Common Hub Gene Expression in Dataset

**Figure 8:**
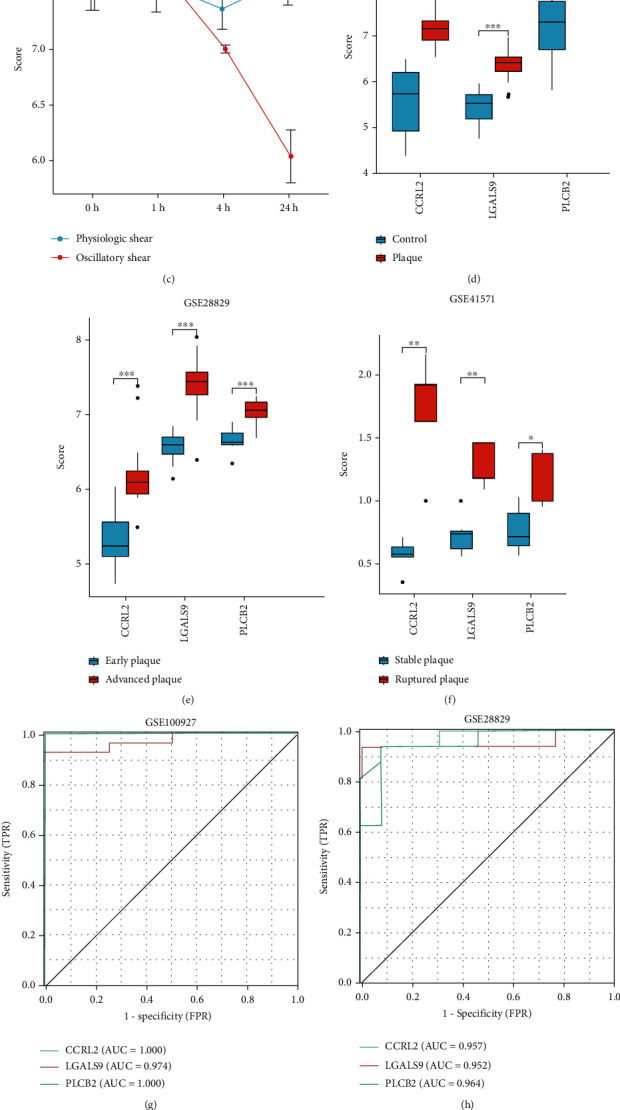
(a–c) The expression levels of *CCRL2*, *LGALS9*, and *PLCB2* in the GSE160611. (d) The expression levels of three common hub genes between the plaque and control tissue in GSE100927. (e) The expression levels of common hub genes between the advanced plaque and early plaque in the GSE28829. (f) The expression levels of common hub genes between ruptured plaque and stable plaque in the GSE41571. (g–i) Receiver operating characteristic (ROC) analysis of these common hub genes in GSE100927, GSE28829, and GSE41571.

To confirm and validate the expression of the three common hub genes in HAECs under different shear stress and atherosclerotic plaque, the expression of the three common hub genes was then validated using GSE160611, GSE100927, GSE28829, and GSE41571. For all three common hub genes, PS and OS conditions resulted in significant changes in gene expression at 3 time points (1, 4, and 24 hours). After 24 hours of physiologic shear stress, the expression levels of *CCRL2* and *LGALS9* were significantly increased, while *PLCB2* shows no significant change (Figures 8(a) and 8(b)). A 24-hour OS force significantly reduced expression of *CCRL2* and *PLCB2* in ECs (Figures 8(a) and 8(c)).

After the application of pulsatile or physiologic shear stress for 24 hours, the expression levels of all three genes showed significant statistical differences (Figures 8(a)–8(c)). The three common hub genes increased in the atherosclerotic plaque compared with control tissue in GSE100927 (Figure 8(d)). Further mRNA analysis in GSE28829 showed that these genes were also increased in advanced plaques compared to early plaques (Figure 8(e)). In ruptured plaques, the expression of the three common hub genes, including *CCRL2*, *LGALS9*, and *PLCB2*, significantly increased compared to stable human plaques, suggesting their correlation with plaque progression (Figure 8(f)).

### 3.7. ROC Curve Analyses of the Common Hub Genes in Atherosclerotic Disease

ROC curve analyses were conducted to assess the ability of common hub genes to distinguish samples of atherosclerotic plaque from normal arterial. Their ROC curves indicated that the expression of *CCRL2* (AUC = 1), *LGALS9* (AUC = 0.974), and *PLCB2* (AUC = 1) could effectively distinguish the atherosclerotic plaque and normal arterial (Figure 8(g)). Moreover, we confirmed the powerful discrimination ability of these three mRNA in GSE28829 with an AUC of 0.957 in *CCRL2*, AUC of 0.952 in *LGALS9*, and AUC of 0.964 in *PLCB2* (Figure 8(h)). They also demonstrated strong discriminatory power for advanced and early atherosclerotic plaques. We further evaluated the diagnostic efficacies of each independent parameter to discriminate the rupture plaque and stable plaque in GSE41571 (Figure 8(i)).

### 3.8. Increased Expression of Common Hub Genes in Macrophage-Derived Foam Cells

**Figure 9:**
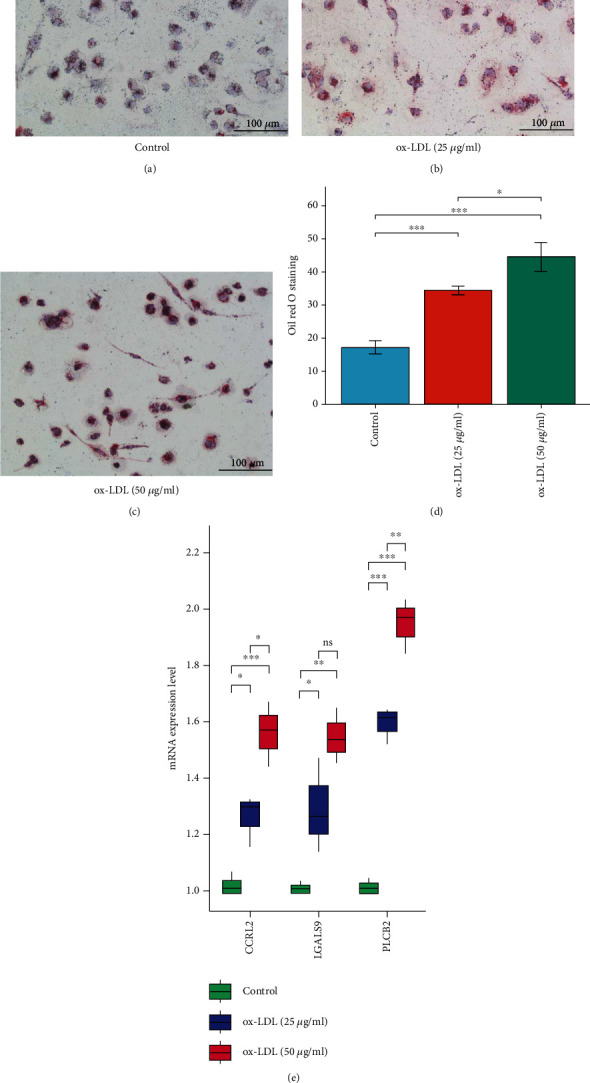
(a–c) Representative Oil Red O staining of macrophage. (d) Quantitative measurement of Oil Red O staining using ImageJ. (e) The expression levels of common hub genes in ox-LDL-induced macrophage.

The results showed that ox-LDL promotes the formation of foam cells from macrophages; Oil Red O staining and quantitative analysis showed the accumulation of lipid in red (Figures 9(a)–9(d)). The RT-qPCR results showed that the mRNA expressions of CCRL2, LGALS9, and PLCB2 were significantly increased, following ox-LDL stimulation in macrophages (Figure 9(e)). Moreover, the results revealed that as the accumulation of lipid increased, the expression level of common hub genes increases (Figures 9(d) and 9(e)).

## 4. Discussion

It is known that vascular ECs first sense variations of the OS, a known activator of ECs that promote the formation and development of atherosclerotic plaque mainly in bifurcated vessels such as carotid arteries [33–35]. In fact, vascular injury and endothelial dysfunction induced by OS are often regarded as a hallmark for AS initiation. Continuous low-grade injury to ESs, induced by disturbed flow at arterial branch points and curvatures, could lead to apoptosis and inflammation, causing endothelial cell dysfunction which was considered critical initiating step in the pathogenesis of AS. Therefore, further studies are necessary to fully understand the potential mechanisms. In the present study, we focused on alterations in EC gene expression that result from OS, which are capable of inducing atherosclerotic plaque progression.

Earlier studies showed that excessively low blood flow shear is able to induce the change of the gene expression pattern of ECs, leading to the progression of atherosclerotic lesions [6, 14]. Studies have reported that shear stress may regulate the growth characteristics of vascular smooth muscle cells by altering the EC and inflammatory regulation and then contribute the formation of atherosclerotic lesions [13, 15]. Based on the above researches, we further studied the specific molecular mechanism of atherosclerotic plaque progression induced by OS using bioinformatics technology. In this study, we analyzed the changes of gene expression profiles of ECs exposed to PS and OS (GSE160611 and GSE103672). Using bioinformatics techniques, we identified three AS-related modules and three shear stress-related modules from GSE28829 and GSE160611 in discovery set and identified 129 significant genes (SET1) from six modules. Differential gene analysis of the validation set yielded 1955 atherosclerosis-related DEGs and 3680 shear stress-related DEGs, from which 476 key genes (SET2) were identified. To search for preventive and therapeutic targets for AS induced by OS, three co-hub genes were screened out: *CCRL2*, *LGALS9*, and *PLCB2*. In addition, the expression of these genes in advanced atherosclerotic plaques was also studied. Compared with their expression levels in control tissues, the expression of three genes was significantly increased in atherosclerotic plaques. ROC analysis demonstrates that the differential expression of these three genes had reliable value in differentiating plaques and even identifying advanced stages. The three promising mRNAs, proposed by this study, could provide some clues to reveal the potential molecular mechanism of OS and AS. These data will also help to predict the clinical deterioration of patients with advanced and ruptured AS plaque and may also provide potential targets for treatment.

Through functional enrichment analysis of SET1 and SET2, we found that, regardless of which genetic screening modality (DEG or WGCNA) was employed, GO analysis showed significant enrichment of “cell adhesion,” “intracellular signal transduction,” “inflammatory response,” “angiogenesis,” and “cytokine-mediated signaling pathway.” The broad similarity of the functional categories of genes of SET1 and SET1 underscored the vital role of these signaling pathways in OS stress-induced atherosclerotic plaque progression.

To further identify hub genes and key signaling pathways, MCODE plug-in of Cytoscape was conducted to identify the densely connected clusters from PPI networks of SET1 and SET2. We then evaluated biological pathways enriched in respective cluster using ClueGO. Genes in cluster 1 and cluster 2 were mainly involved in cell migration, especially vascular ECs. Endothelial cell damage caused by OS is the first step in the formation of atherosclerotic lesions [36]. Endothelial cell proliferation and migration are of great importance in the pathological process of early atherosclerotic plaque formation. Migration of leukocytes and monocytes into the endothelial cell layer is an important event in the pathogenesis of AS [37]. Earlier studies showed that strong shear stress resulting from fluid flow under normal conditions would impede leukocyte adhesion and subsequent migration; it is well documented that localized OS stress would facilitate this process and promotes atherosclerotic plaque development, although these arterial shear sizes would still be larger than venous wall shear sizes [38, 39]. The gene in cluster 3 is mainly focus mononuclear cell and T cell migration. Despite the macrophages are the key cells in AS, T cell subpopulations are thought to be important in triggering atherosclerotic inflammatory processes [40, 41]. Additionally, T cell-mediated immune function imbalance plays an important role in the pathological process of AS [42]. T helper (Th) 1 profile is the most abundant pathogenic T cells in AS which plays a proatherogenic role by activating monocytes/macrophages and dendritic cells (DCs) through secretion of proinflammatory cytokines [43]. The role of Th2 in AS is still controversial and relates to the site and stage of the atherosclerotic plaque [44]. Our findings provide a further demonstration of the regulation of the OS on cell migration (endothelial cell, mononuclear cell, and T cell) and the consequences for the formation and progression of early atherosclerotic plaques.

The three co-hub genes (*CCRL2*, *LGALS9*, and *PLCB2*) identified from the clusters are likely to be key genes involved in these regulatory processes and novel therapeutic targets to prevent or treat AS. Interestingly, when OS or PS force was applied for 24 hours, the common hub genes in OS shear force-induced ECs were expressed at much lower levels than those in PS-induced ECs, and this difference was statistically significant. On the other hand, we detected that the expression of three common hub genes appears to correlate with the progression of atherosclerotic plaques, and its expression increased with disease progression.

*CCRL2*, originally cloned from LPS-activated macrophages, is a 7-transmembrane domain nonsignaling atypical receptor with functional similarity to the atypical chemokine receptor family [45, 46]. Studies have shown that *CCRL2*, which is widely expressed by endothelial and epithelial cells and by a variety of leukocytes, including macrophages, dendritic cells, and neutrophils, regulates immune responses under several inflammatory conditions [45, 47]. Previous studies have shown that *CCRL2* sequesters secreted chemerin, promptly concentrate it, and conjugate with ChemR23-expressing dendritic cells (DCs) and promote the transmigration of DCs across the endothelial cell monolayer [46, 47]. *CCRL2*, as a receptor of chemerin, binding to chemerin activates the nuclear factor kappa B (NF-*κ*B) and Janus kinase (JAK)/STAT pathways in ECs and increases VCAM-1 expression, promoting lymphocyte-EC adhesion [48]. In this study, the hub gene enrichment analysis results indicated that “regulation of blood vessel endothelial cell migration” and “regulation of lymphocyte migration” signaling pathways were significantly enriched in hub genes. Combining the results of the enrichment analysis with earlier published *CCRL2* studies, we surmise that *CCRL2* regulates monocyte adhesion to ECs, a key step in the initiation and progression of abnormal ESS-induced AS.

LGALS9 (galectin-9) was firstly characterized as a chemoattractant for eosinophils. Recent lines of evidence have implicated that *LGALS9* is a multifaceted immune regulator in multiple cell types [49]. *LGALS9*, as a ligand of T cell immunoglobulin mucin 3 (Tim-3), can regulate the function of a variety of AS-associated immune cells, including effector T cells and macrophages, especially regulatory T cells [49]. By enhancing Foxp3 expression, *LGALS9* promoted the differentiation of naïve T cells into regulatory T (Treg) cells, which was also supported by the study that the number of Tregs was decreased in Gal-9 knockout mice [50, 51]. Expression of Gal-9 by *LGALS9* on the cell surface may enhance the suppressive activity of these cells on Th1 and Th17 cells, exerting a proatherogenic effect [52]. Recent studies have shown that *LGALS9* signaling regulates autoimmunity through Tim-3, accompanied by promoting macrophage phenotypic transition to anti-inflammatory type, increasing the number of Tregs, and decreasing in numbers of effector T cells [53, 54]. Tim-3 exhibited an antiatherosclerotic effect through various mechanisms, including NF-*κ*B inhibition and reduction of vascular smooth muscle cell proliferation and migration [55].

Previous studies indicated that *LGALS9* has an important role in AS. Our study shows that *LGALS9* is closely associated with cell migration which agrees with recent studies by O'Brien et al., who pointed out that *LGALS9* induces monocyte migration and significantly increases inflammation [56]. The results of enrichment analysis of three densely connected clusters suggested that cell migration may be an important mechanism of ESS-induced AS. As a hub gene screened from densely connected clusters, the increased expression of *LGALS9* may play an important role in ESS-induced AS by regulating the migration of cells.

*PLCB2* is a protein coding gene, and its downstream protein phospholipase C (PLC)-*β*2 plays a major role in platelet activation [57]. PLC-*β*2 catalyzes the hydrolysis of phosphatidylinositol 4,5-bisphosphate to the second messenger inositol 1,4,5-trisphosphate (IP3) and diacylglycerol and a critical regulator role in platelet responses to activation of G*α*q-coupled receptors by thromboxane A2, thrombin, and ADP [58]. NF-*κ*B has a regulatory effect on the transcription of this gene, and its protein product plays an important role in platelet responses. Similar finding was confirmed by Mao et al.: knockdown of *NF-κB* p65 subunit by siRNA decreased *PLCB2* expression, and *PLCB*2 expression was increased by *p6*5 overexpression [58]. NF-*κ*B appears to play a central role in the proinflammatory activation in atherogenesis, and the role of platelet activation in AS has been highlighted since increased platelet production accelerates atherogenesis [59, 60]. However, to the best of our knowledge, no *PLCB2* studies on AS have been reported so far at all. Our data preliminarily show that *PLCB2* plays an essential role in AS and abnormal ESS, and further studies are needed to elucidate its mechanism of action.

We think that a series of genome-wide, unbiased screens for identifying hub genes would be of considerable value for revealing mechanisms and potential therapeutic targets. Our present findings have demonstrated that *CCRL2*, *LGALS9*, and *PLCB2* play vital roles in abnormal ESS and AS. These genes may warrant as valuable targets for prevention and treatment of AS induced by abnormal ESS.

This study has some limitations. First, due to the small sample size of datasets, larger studies are required to confirm our findings. Second, different sample sources and detection microarray platforms may contribute to some of the differences in gene expression. In addition, GSE160611 and GSE103672 contain only sequencing results from in vitro cultured ECs, and no clinical specimens were obtained for further validation. In the future, to verify our hypothesis, more research that includes larger samples, randomized trial designs, further mechanistic studies, and even more clinical trials is needed.

## 5. Conclusions

This preliminary study provides evidence linking altered ESS to AS and atherosclerotic plaques. Specifically, we identified *CCRL2*, *LGALS9*, and *PLCB2* as key genes associated with abnormal ESS and AS and initially explored the roles of these genes in endothelial dysfunction and AS, thus highlighting the possibility of new preventive and therapeutic strategies for AS by targeting these genes. These genes were correlated with cell migration, thereby promoting plaque progression, and it may even contribute to plaque rupture as it facilitates the recruitment of immune cells. Whereas the role played by *PLCB2* in AS is not clear, we speculate that its expression may affect AS by regulating platelet activation.

Hemodynamic shear stress is essential for endothelial homeostasis under physiological or pathological conditions. The impact of abnormal shear stress on the ECs and its further impact on AS are a very complex multifactorial process, and our study preliminarily speculates on the mechanism and key genes involved through the analysis of sequencing data. However, the specific signaling pathways and mechanisms involved require further studies to demonstrate.

## Figures and Tables

**Table 1:** GEO dataset information.

GEO ID	Platform	Samples	Group
GSE28829	GPL570	Atherosclerotic plaque (16 advanced and 13 early)	Discovery set
GSE160611	GPL20301	HAECs (pulsatile shear and oscillatory shear)	Discovery set
GSE100927	GPL17077	Carotid artery (29 plaque and 11 control)	Validation set
GSE103672	GPL11154	HUVECs (pulsatile shear and oscillatory shear)	Validation set

## Data Availability

Datasets used in this study were obtained from open public databases (https://www.ncbi.nlm.nih.gov/geo/), but the datasets analyzed in this study are available from the corresponding authors on request.

## Abbreviations

AS: Atherosclerosis
ECs: Endothelial cells
ESS: Endothelial shear stress
PS: Pulsatile shear
OS: Oscillatory shear
TCFA: Thin-cap fibroatheroma
GEO: Gene Expression Omnibus
HAECs: Human aortic endothelial cells
ST: Static condition
HUVECs: Human umbilical vein endothelial cells
WGCNA: Weighted gene coexpression network analysis
DEG: Differentially expressed genes
GO: Gene Ontology
KEGG: Kyoto Encyclopedia of Genes and Genomes
BP: Biological process
CC: Cellular component
MF: Molecular function
PPI: Protein-protein interaction
MCODE: Molecular complex detection
ROC: Receiver operating characteristic
AUC: Area under the curve
DCs: Dendritic cells
PLC-*β*2: Phospholipase C-*β*2
NF-*κ*B: Nuclear factor kappa B.

## Supplementary Materials

Supplementary Materials All supplementary diagrams are included in the “Supplementary Materials” file. Supplementary Table 1: genes in SET1 and SET2. Supplementary Table 2: significantly enriched pathways in SET1: the term represents the name of enriched pathways, and count represents the number of genes enriched in the pathway. Supplementary Table 3: significantly enriched pathways in SET2. Supplementary Table 4: genes included in cluster 1, cluster 2 and cluster 3. Supplementary Table 5: primer sequences of LGALS9, PLCB2, CCRL2, and GAPDH.
